# Associations of Plasma Phospholipid and Dietary Alpha Linolenic Acid With Incident Atrial Fibrillation in Older Adults: The Cardiovascular Health Study

**DOI:** 10.1161/JAHA.112.003814

**Published:** 2013-02-22

**Authors:** Amanda M. Fretts, Dariush Mozaffarian, David S. Siscovick, Susan R. Heckbert, Barbara McKnight, Irena B. King, Eric B. Rimm, Bruce M. Psaty, Frank M. Sacks, Xiaoling Song, Donna Spiegelman, Rozenn N. Lemaitre

**Affiliations:** 1 Cardiovascular Health Research Unit, Department of Medicine, University of Washington, Seattle, WA (A.M.F., D.S.S., B.M.P., R.N.L.); 2 Department of Biostatistics, University of Washington, Seattle, WA (B.M.K.); 3 Department of Health Services, University of Washington, Seattle, WA (B.M.P.); 4 Department of Epidemiology, University of Washington, Seattle, WA (D.S.S., S.R.H., B.M.P.); 5 Public Health Sciences Division, Fred Hutchinson Cancer Research Center, Seattle, WA (X.S.); 6 Department of Internal Medicine, University of New Mexico, Albuquerque, NM (I.B.K.); 7 Department of Epidemiology, Harvard School of Public Health, Boston, MA (D.M., E.B.R., D.S.); 8 Department of Biostatistics, Harvard School of Public Health, Boston, MA (D.S.); 9 Department of Nutrition, Harvard School of Public Health, Boston, MA (D.M., E.B.R., F.M.S.); 10 Cardiovascular Medicine, Brigham and Women's Hospital and Harvard Medical School, Boston, MA (D.M., F.M.S.); 11 Channing Laboratory, Brigham and Women's Hospital and Harvard Medical School, Boston, MA (D.M., E.B.R., F.M.S.)

**Keywords:** atrial fibrillation, epidemiology, fatty acids, nutrition

## Abstract

**Background:**

Few studies have examined the relationship of α‐linolenic acid (ALA 18:3n‐3), an intermediate‐chain essential n‐3 polyunsaturated fatty acid derived from plants and vegetable oils, with incident atrial fibrillation (AF).

**Methods and Results:**

The study population included participants from the Cardiovascular Health Study, a community‐based longitudinal cohort of adults aged 65 or older, free of prevalent coronary heart disease and atrial fibrillation. We assessed the associations of plasma phospholipid and dietary ALA with incident AF using Cox regression. The biomarker analysis comprised a total of 2899 participants, and the dietary analysis comprised 4337 participants. We found no association of plasma phospholipid ALA and incident AF. Comparing each of the second, third, and fourth quartiles to the lowest quartile, the hazard ratios for AF were 1.11 (95% CI, 0.90 to 1.37), 1.09 (95% CI, 0.88 to 1.35), and 0.92 (95% CI, 0.74 to 1.15), after adjustment for age, sex, race, clinic, education, smoking, alcohol, body mass index, waist circumference, diabetes, heart failure, stroke, treated hypertension, and physical activity (*P* trend=0.48). When dietary ALA was considered the exposure of interest, results were similar.

**Conclusions:**

Results from this prospective cohort study of older adults indicate no association of plasma phospholipid or dietary ALA and incident AF.

## Introduction

Atrial fibrillation (AF) is the most common chronic arrhythmia in the United States. According to results from the Framingham Heart Study, the lifetime risk of developing AF is 26% in men and 23% in women.^1^ Risk of AF increases with age, and the burden of disease is particularly high among individuals 65 years of age or older.^2^ In 2005, there were more than 3 million individuals in the United States with diagnosed AF, and the prevalence of AF is projected to reach more than 7.5 million by 2050 as the population ages.^3^ Because AF is associated with significant morbidity, including stroke, heart failure, and mortality, and there are limited treatment options for AF, primary prevention of AF is essential.^4–6^

Several studies indicate that long‐chain n‐3 fatty acids derived from seafood, particularly eicosapentaenoic acid (EPA) and docosahexaenoic acid (DHA), are associated with a lower risk of AF.^7–9^ However, little is known about the relationship of α‐linolenic acid (ALA 18:3n‐3), an intermediate‐chain essential n‐3 polyunsaturated fatty acid derived from plants and vegetable oils, with incident AF.^8^ Major dietary sources of ALA include flaxseed, canola and soybean oils, processed foods that contain these oils such as salad dressing and mayonnaise, and walnuts.^10^ Because plant‐derived fatty acids are lower in cost and have greater worldwide availability than seafood, and at least some experimental studies suggest possible physiologic benefits of ALA,^11–13^ a better understanding of the relationship of ALA with incident AF is of public health importance.

Estimation of dietary ALA is challenging because it is present in many foods and depends on food oil composition. Plasma phospholipid ALA, an objective biomarker of dietary ALA that reflects both diet and metabolism, is a measure of circulating ALA over the past 4 to 8 weeks. The purpose of this study was to examine the association of plasma phospholipid and dietary ALA with incident AF in the Cardiovascular Health Study (CHS), a community‐based longitudinal cohort of adults aged 65 or older. We hypothesized that higher levels of plasma phospholipid and dietary intake of ALA are associated with a lower risk of AF among older adults.

## Methods

### Study Population

The CHS is a population‐based longitudinal study of cardiovascular disease among adults aged 65 years or older from 4 US communities (Forsyth County, NC; Sacramento County, CA; Washington County, MD; Allegheny County, PA). Details of the study design and sampling methods have been reported previously.^14^ Medicare eligibility lists were used to randomly select and enroll ambulatory and noninstitutionalized adults aged 65 years or older in the study. In 1989–1990, 5201 participants enrolled in the study and an additional 687 participants (predominantly African American) enrolled in 1992–1993. In total, 57% of randomly sampled adults agreed to participate in the study. Participants were followed by annual clinic visits with interim phone calls for the first 10 years of the study and then by biannual phone contact thereafter. The institutional review board for each site approved the study, and written informed consent was obtained from all participants at enrollment.

For the plasma phospholipid analysis, we excluded participants with AF (n=313), those with coronary heart disease (n=809), and those missing the plasma phospholipid ALA measure (n=1867) at the time of the 1992–1993 blood draw. The study population for the analysis comprised 2899 persons in total. For the dietary ALA analyses, participants with prevalent AF (n=194), with a history of coronary heart disease (n=1122), or missing diet measurement (n=235) at the time of the first diet assessment (1989–1990 for the old cohort or 1995–1996 for the new cohort) were excluded from analyses. The analytic cohort for the dietary ALA analysis comprised the remaining 4337 participants.

### Data Collection

Annual clinic examinations included a standardized interview, physical examination, laboratory evaluations, and diagnostic testing, including 12‐lead electrocardiograms (ECGs). Information regarding medical history, education, smoking, alcohol consumption, and cardiovascular risk factors was collected at the interview. Blood samples were collected after a 12‐hour overnight fast and stored at −70°C.

### Plasma Phospholipid ALA Measurement

ALA was measured at the Fred Hutchinson Cancer Research Center (Seattle, WA) using stored samples from 1992–1993. ALA is expressed as percentage of total fatty acids. Total lipids were extracted from plasma using the methods of Folch, as described previously.^15^ Phospholipids were separated from neutral lipids using 1‐dimensional thin‐layer chromatography. To prepare fatty acid methyl esters, phospholipid fractions were directly transesterified using the Lepage and Roy method.^16^ Gas chromatography was used to separate individual fatty acids from methyl esters, as previously described (Agilent 5890 Gas Chromatograph flame ionization detector, Agilent Technologies, Palo Alto, CA; fused silica capillary column SP‐2560 [100 m×0.25 mm, 0.2 μm], Supelco Belefonte, PA; initial 160°C for 16 minutes, ramp 3°C/minute to 240°C, hold 15 minutes).^17^ Laboratory interassay coefficient of variation was 3.1% for ALA.^18^

### Dietary ALA Measurement

Food frequency questionnaires (FFQs) were administered in 1989–1990 and 1995–1996 to measure usual dietary intake. The 1989–1990 FFQ was a picture version of the National Cancer Registry FFQ and included 99 food items.^19^ In 1995–1996, the Willett 131‐item semiquantitative FFQ was administered.^20^ Both these questionnaires have been shown to be valid and reliable when compared with repeated 24‐hour dietary recalls or 1‐week dietary records.^19–20^ Each participant was asked how often, on average, specified foods had been consumed during the past year. To obtain a measure of dietary ALA intake, the frequency response for each food on the FFQ was multiplied by the dietary ALA content of the food and then summed for all foods. For the purposes of this analysis, dietary intake of ALA is expressed as percentage of total fat. Participants entered the analysis at the time of their first dietary assessment (1989–1990 or 1995–1996). We cumulatively updated dietary ALA intake among participants who completed both the 1989–1990 and the 1995–1996 FFQ and did not develop coronary heart disease (CHD) during 1989–1996 (n=2436). For participants who developed CHD during 1989–1996 (n=422), only the 1989–1990 measure of dietary ALA was used for analyses. We chose not to update dietary ALA intake among participants who developed CHD during follow‐up because diagnosis may have influenced diet or risk of AF. For participants who enrolled in the CHS during 1992–1993 (n=350), the 1995–1996 diet measure was used for analyses.

### Atrial Fibrillation Assessment

Incident AF, including atrial flutter, was identified from 12‐lead ECGs performed annually until 1999 or from hospital discharge diagnoses (ICD‐9 codes 427.3, 427.31, 427.32)^21^ through June 30, 2008. Review of medical records of a subsample of participants in the CHS with a hospital discharge code for AF indicates that the positive predictive value of AF identification through hospital diagnosis codes is 98.6%.^22^ In addition, among 819 CHS participants who underwent a 24‐hour Holter monitor assessment at the 1994–1995 exam, only 1 study participant had sustained or intermittent AF identified via the Holter monitor that was not identified by either ECG or hospital discharge diagnosis codes.^7^ In total, study participants were followed for up to 16 years for the plasma phospholipid ALA analyses and up to 19 years for the dietary ALA analyses for development of AF.

### Statistical Analyses

All statistical analyses were conducted using STATA version 10.0 (Stata Corp, College Station, TX). The associations of incident AF with plasma phospholipid and dietary ALA were assessed both linearly and categorically (comparing each of the top 3 quartiles to the lowest) using multiple Cox regression models. Death from any cause and loss to follow‐up were considered censoring events. The proportional hazards assumption for both plasma and dietary ALA was evaluated using Schoenfeld's residuals. To avoid false associations of nutrients with disease risk because of confounding by total reported energy intake, all dietary analyses were adjusted for total energy intake.^23^

Two levels of adjustment were used to examine the associations of ALA with incident AF. Minimal adjustments included age and sex (and total caloric intake for dietary analyses) at the time of the plasma phospholipid ALA measure (or the first diet measure for dietary analyses). Additional adjustments included race (white or African American), clinic (Bowman Gray, Davis, Hopkins, Pittsburg), education (no high school, high school/vocational school, college), smoking (never, past, current), diabetes (yes/no), history of heart failure (yes/no), history of stroke (yes/no), body mass index (BMI; kg/m^2^), waist circumference (cm), physical activity (kcal/week), alcohol use (drinks/week), and treated hypertension (yes/no) at the time of the plasma phospholipid ALA measure (or the first diet measure for dietary analyses). Because linoleic acid (LA), a major dietary polyunsaturated fatty acid that competes with ALA for elongation into longer‐chain n‐3 and n‐6 fatty acids, may overwhelm the association of ALA and AF, we additionally adjusted for LA in sensitivity analyses. In addition, exposure misclassification may increase with increasing duration of follow‐up because of changes in diet or metabolism over time, and we performed sensitivity analyses censoring 5 and 10 years after the first blood draw.

We examined the potential interaction of ALA with sex and age to investigate whether these factors modify the association of ALA and AF. In addition, because LA may prevent the elongation/desaturation of ALA into long‐chain n‐3 fatty acids,^24^ we examined the interaction of LA and ALA on risk of AF. Because conversion of ALA to EPA and DHA is catalyzed by the delta‐6‐desaturase enzyme and genetic variability in the delta‐6‐desaturase gene (*FADS2*) may affect the conversion,^25^ we also examined the interaction between the *FADS2* genotype and ALA. Wald's tests were used to evaluate the statistical significance of the multiplicative interaction term for each factor, with ALA modeled linearly. Because EPA and DHA have been shown to prevent the desaturation/elongation of ALA,^26^ it is possible that the effect of ALA on AF may differ among those with high versus low fish intake. In sensitivity analyses, we stratified the analyses at the 25th percentile of fish intake (0.55 servings/day).

Missing covariates (<2% for all covariates, except alcohol intake, for which it was 4.0%) were imputed by multiple imputations using data on age, sex, smoking, education, race, BMI, physical activity, self‐reported health status, and diabetes at the time of the plasma phospholipid ALA measure (or the first diet measure for dietary analyses).

## Results

Among the 2899 CHS participants who made up the analytic cohort, 63.6% were female, and the median age at the 1989–1990 examination was 74 years (range, 65 to 98 years). Plasma phospholipid ALA represented <1% of total fatty acids (median, 0.14% total fatty acids; range, 0.05% to 0.47% total fatty acids). Dietary ALA (percent total fat) and plasma phospholipid ALA were modestly correlated (*r*=0.18). Plasma phospholipid ALA was positively correlated with plasma phospholipid LA (*r*=0.26) and dietary LA (*r*=0.15) and negatively correlated with plasma phospholipid arachidonic acid (AA) (*r*=−0.37), but it was poorly correlated with plasma phospholipid EPA, DHA, and DPA (*r*=0.06) and fish intake (*r*=0.07).

Characteristics of the study participants in 1992–1993 according to quartile of plasma phospholipid ALA are shown in Table 1. Participants with higher plasma phospholipid ALA were more likely to be female and white, consumed slightly more alcohol per week, reported higher levels of education, had higher levels of plasma phospholipid and reported dietary LA, were less likely to smoke, take lipid‐lowering medication, or report a history of stroke, heart failure, or diabetes, and had lower levels of plasma phospholipid AA, BMI, and waist circumference when compared with participants with lower levels of plasma phospholipid ALA. There were no differences in treated hypertension, aspirin use, plasma phospholipid long‐chain n3‐fatty acid levels, or reported fish intake according to quartile of plasma phospholipid ALA.

**Table 1.** Characteristics of 2899 Adults Aged 65+ in 1992–1993 According to Plasma Phospholipid ALA

	Q1	Q2	Q3	Q4
ALA, % total FA, median	0.10	0.13	0.16	0.21
Range	0.05, 0.11	0.11, 0.14	0.14, 0.18	0.18, 0.47
n	738	711	702	748
Age, y	74.5 (5.0)	74.7 (5.2)	74.7 (5.2)	75.0 (5.2)
Sex, % male	45.1	36.4	34.3	29.5
Race, % white	82.2	86.6	87.5	90.4
Education >high school, %	34.7	34.7	39.5	40.8
Current smoking, %	11.7	11.0	8.0	7.6
Diabetes mellitus, %	17.3	16.7	13.4	10.7
History of stroke, %	5.0	4.6	3.1	2.5
Heart failure, %	2.2	2.7	2.1	1.7
Treated hypertension, %	36.3	38.1	35.0	36.5
Aspirin >2 days in 2 weeks, %	31.1	30.3	31.9	28.6
Lipid‐lowering medication, %	8.0	6.2	3.7 (0.2)	4.7 (0.2)
Body mass index, kg/m^2^	27.7 (5.1)	27.0 (4.6)	26.6 (4.5)	25.7 (4.4)
Waist circumference, cm	100.1 (13.7)	97.5 (12.8)	96.4 (13.2)	94.1 (13.1)
Physical activity, kcal/week	1462.4 (1712.9)	1373.7 (1650.4)	1338.3 (1564.4)	1615.2 (1916.0)
Alcohol, drinks/week	1.6 (3.9)	1.8 (4.8)	2.2 (5.4)	2.6 (5.3)
Fish intake, servings/week	1.5 (1.4)	1.5 (1.2)	1.5 (1.4)	1.7 (1.5)
Estimated dietary LA, % total fat	0.18 (0.04)	0.19 (0.05)	0.19 (0.05)	0.20 (0.05)
PP EPA+DHA+DPA, % total FA	4.4 (1.2)	4.5 (1.3)	4.5 (1.2)	4.5 (1.2)
PP LA, % total FA	18.8 (2.4)	19.4 (2.4)	19.9 (2.3)	20.6 (2.6)
PP AA, % total FA	12.0 (1.9)	11.4 (1.8)	11.0 (1.7)	10.1 (1.9)

Data presented as mean (SD) or else percentage. ALA indicates α‐linolenic acid; FA, fatty acid; LA, linoleic acid; PP, plasma phospholipid; EPA, eicosapentaenoic acid; DHA, docosahexaenoic acid; DPA, docosapentaenoic acid; AA, arachidonic acid.

During 29 863 person‐years of follow‐up, there were 707 cases of incident AF. We found no association of plasma phospholipid ALA and incident atrial fibrillation. Comparing each of the second, third, and fourth quartiles to the lowest quartile, the hazard ratios for atrial fibrillation were 1.11 (95% CI, 0.90 to 1.37), 1.09 (95% CI, 0.88 to 1.35) and 0.92 (95% CI, 0.74 to 1.15), after adjustment for age, sex, race, clinic, education, smoking, alcohol, BMI, waist circumference, diabetes, heart failure, stroke, treated hypertension, and physical activity (*P* trend=0.48) (Table 2). Modeling ALA linearly, additionally adjusting for linoleic acid, or restricting analyses to the first 7 years of follow‐up did not materially alter results (data not shown). There were also no statistically significant interactions between plasma phospholipid ALA and age, sex, linoleic acid, or *FADS* genotype when assessing risk of AF (smallest *P* for interaction=0.13). When dietary ALA was considered the exposure of interest, results were similar (Table 3).

**Table 2.** Hazard Ratio for Incident Atrial Fibrillation According to Plasma Phospholipid ALA Among 2899 Adults Aged 65+

	Quartile				*P* Value Trend
	I	II	III	IV	
Person‐years	7616	7156	7170	7921	
Number of cases	178	182	177	170	
Hazard ratio (95% CI)					
Age‐ and sex‐adjusted	1.0 (ref)	1.15 (0.93 to 1.41)	1.11 (0.90 to 1.37)	0.95 (0.77 to 1.18)	0.60
Multivariate model*	1.0 (ref)	1.11 (0.90 to 1.37)	1.09 (0.88 to 1.35)	0.92 (0.74 to 1.15)	0.48

ALA indicates α‐linolenic acid; CI, confidence interval; BMI, body mass index.

* Also adjusts for race, clinic, education, smoking, diabetes, history of heart failure, history of stroke, BMI, waist circumference, physical activity, alcohol use, and treated hypertension.

**Table 3.** Hazard Ratio for Incident Atrial Fibrillation According to Dietary ALA Intake* Among 4337 Adults aged 65+

	Quartile				*P* Value Trend
	I	II	III	IV	
ALA, % total fat intake, median	1.5	1.9	2.2	2.8	
Range	0.8, 1.7	1.8, 2.0	2.1, 2.4	2.5, 4.8	
Person‐years	11 118	13 362	13 683	11 900	
Number of cases	272	277	271	269	
Hazard ratio (95% CI)					
Age‐and‐sex‐adjusted	1.0 (ref)	0.87 (0.73 to 1.03)	0.89 (0.75 to 1.06)	1.05 (0.88 to 1.25)	0.55
Multivariate model*	1.0 (ref)	0.87 (0.74 to 1.04)	0.90 (0.75 to 1.07)	1.06 (0.89 to 1.27)	0.48

ALA indicates α‐linolenic acid; CI, confidence interval; BMI, body mass index.

* ALA expressed as percentage of total fat.

* Also adjusts for race, clinic, education, smoking, diabetes, history of heart failure, history of stroke, BMI, waist circumference, physical activity, alcohol use, and treated hypertension.

Associations of ALA levels with AF incidence were generally similar in analyses stratified by sex (Tables 4 and 5). In addition, the associations of ALA levels with n‐6 and n‐3 fatty acids (LA, AA, total EPA+DHA+DPA) were similar in men and women (Table 6). Restricting analyses to subjects with low fish consumption (defined as <0.55 servings/week) or high fish consumption (≥0.55 servings/week) did not alter the results.

**Table 4.** Hazard Ratio for Incident Atrial Fibrillation According to Plasma Phospholipid ALA Among 2899 Adults Aged 65+

Quartile	Female				*P* Trend	Male				*P* Value Trend
	I	II	III	IV		I	II	III	IV	
Person‐years	4972	4886	4992	5180		2800	2232	2261	2540	
Number of cases	100	110	99	103		82	76	70	67	
Hazard ratio (95% CI)										
Age‐adjusted	1.0 (ref)	(0.88 to 1.52)	0.98 (0.74 to 1.30)	0.99 (0.75 to 1.30)	0.64	1.0 (ref)	1.09 (0.80 to 1.49)	1.05 (0.77 to 1.46)	0.85 (0.61 to 1.33)	0.33
Multivariate model*	1.0 (ref)	1.08 (0.82 to 1.43)	0.95 (0.71 to 1.27)	0.95 (0.71 to 1.26)	0.53	1.0 (ref)	1.10 (0.80 to 1.51)	1.07 (0.77 to 1.48)	0.81 (0.58 to 1.31)	0.42

ALA indicates α‐linolenic acid; CI, confidence interval; BMI, body mass index.

* Also adjusts for race, clinic, education, smoking, diabetes, history of heart failure, history of stroke, BMI, waist circumference, physical activity, alcohol use, and treated hypertension.

**Table 5.** Hazard Ratio for Incident Atrial Fibrillation According to Dietary ALA* Intake among 4337 Adults Aged 65+

Quartile	Female				*P* Trend	Male				*P* Value Trend
	I	II	III	IV		I	II	III	IV	
Person‐years	5561	8137	9826	8898		5558	5525	3856	3002	
Number of cases	133	146	174	179		112	143	106	96	
Hazard ratio (95% CI)										
Age‐adjusted	1.0 (ref)	0.77 (0.61 to 1.07)	0.74 (0.59 to 1.05)	0.98 (0.78 to 1.22)	0.77	1.0 (ref)	0.97 (0.68 to 1.38)	1.02 (0.72 to 1.45)	0.92 (0.63 to 1.34)	0.76
Multivariate model*	1.0 (ref)	0.78 (0.62 to 1.08)	0.75 (0.60 to 1.07)	1.00 (0.79 to 1.26)	0.90	1.0 (ref)	0.94 (0.65 to 1.35)	0.98 (0.68 to 1.42)	0.87 (0.59 to 1.30)	0.59

ALA indicates α‐linolenic acid; CI, confidence interval; BMI, body mass index.

* ALA expressed as percent of total fat.

* Also adjusts for race, clinic, education, smoking, diabetes, history of heart failure, history of stroke, BMI, waist circumference, physical activity, alcohol use, and treated hypertension.

**Table 6.** Characteristics of 2899 Adults Aged 65+ in 1992–1993 According to Plasma Phospholipid ALA

Quartile	Women				Men			
	I	II	III	IV	I	II	III	IV
ALA, % total fatty acids, mean	0.10	0.13	0.16	0.23	0.09	0.12	0.15	0.21
Range	0.05, 0.12	0.12, 0.15	0.15, 0.18	0.18, 0.44	0.05, 0.11	0.11, 0.13	0.14, 0.17	0.17, 0.48
n	456	463	455	471	289	251	250	264
Fish intake, servings/week	1.7 (1.5)	1.6 (1.3)	1.6 (1.4)	1.8 (1.6)	1.3 (1.0)	1.3 (1.2)	1.5 (1.2)	1.5 (1.5)
Estimated dietary LA, % total FA	0.19 (0.04)	0.20 (0.05)	0.20 (0.05)	0.21 (0.05)	0.18 (0.04)	0.18 (0.04)	0.19 (0.04)	0.19 (0.04)
PP EPA+DHA+DPA, % total FA	4.4 (1.2)	4.5 (1.4)	4.4 (1.1)	4.6 (1.4)	4.3 (1.2)	4.4 (1.2)	4.6 (1.4)	4.5 (1.4)
PP LA, % total FA	18.4 (2.3)	19.2 (2.3)	19.7 (2.3)	20.4 (2.4)	19.2 (2.3)	19.9 (2.5)	20.4 (2.3)	21.1 (2.7)
PP AA, % total FA	12.3 (2.0)	11.5 (1.7)	11.2 (1.7)	10.1 (1.8)	11.7 (1.8)	11.1 (1.8)	10.7 (1.7)	10.0 (1.8)

Data presented as mean (SD) or else percentage. ALA indicates α‐linolenic acid; LA, linoleic acid; FA, fatty acid; PP, plasma phospholipid; EPA, eicosapentaenoic acid; DHA, docosahexaenoic acid; DPA, docosapentaenoic acid; AA, arachidonic acid.

## Discussion

Results from this prospective cohort study of older adults indicate no association of plasma phospholipid or dietary ALA and incident AF. To date, only 1 other study has examined the association of circulating levels of ALA and incident AF in humans.^8^ In that study of Finnish men (mean age at baseline, 53 years), serum ALA was not associated with hospital‐diagnosed AF during 18 years of follow‐up. In addition, the study found no evidence of effect modification by fish intake (high intake versus low intake). In other words, although the populations (middle‐aged versus elderly) and outcome ascertainment (hospital diagnosis versus hospitalized and nonhospitalized cases) differed between the 2 studies, the findings were consistent and showed no association of ALA with incident AF in humans.

In our analysis, plasma phospholipid ALA was only modestly correlated with dietary ALA measured from the FFQs (*r*=0.18). This is not surprising, as measurement of dietary intake of ALA is prone to error because ALA is present in varying amounts in many foods and oils. In addition, it is unknown if plasma phospholipid ALA is a good marker of dietary ALA intake. Because dietary ALA has been shown to be metabolized shortly after consumption or elongated and desaturated to form EPA and DHA in limited amounts, ALA that accumulates in serum may not be a good marker of dietary ALA intake.^27^

Results from observational studies that have examined the association of dietary intake of fish or n‐3 polyunsaturated fatty acids (using dietary questionnaires) with incident AF are conflicting.^7,28–31^ On the other hand, circulating levels of long‐chain n‐3 fatty acids have been consistently associated with a lower risk of AF.^8,32^ In CHS in particular, consumption of broiled or baked fish, particularly fatty fish such as tuna^7^ and plasma phospholipid total long‐chain omega‐3 fatty acids^32^ were both associated with a lower risk of AF. By comparison, findings from the CHS indicate that the n‐3 fatty acid from plants, ALA, was not associated with AF risk.

This study has several strengths. The CHS is a population‐based multicenter cohort study in adults aged 65 years or older—a population at high risk for AF. Unlike the previous study of ALA and AF, which relied on hospital diagnoses to capture AF cases, the CHS used both ECGs, performed annually until 1999, and hospital discharge diagnoses to identify incident cases. This may lower the possibility of misclassification of AF. Other strengths of this study include the availability of detailed demographic and clinical data from the annual study visits to adjust for a variety of potential confounders and the use of an objective measure of ALA. Moreover, the use of both plasma phospholipid and diet ALA measures in this analysis allowed us to explore the associations of both diet and metabolism (plasma phospholipid ALA) and diet (dietary ALA) on risk of AF.

This analysis also has limitations. Plasma phospholipid ALA was only measured at 1 exam (1992–1993), and we were unable to account for potential changes in circulating levels of ALA from fluctuations in diet and metabolism that occurred during the 16 years of follow‐up. However, restricting analyses to the first 5 or 10 years of follow‐up did not materially alter estimated hazard ratios. Intake of dietary ALA was based on responses to an FFQ, and some participants might not have accurately recalled dietary information such as specific foods consumed, frequency, or portion sizes, thereby limiting our ability to accurately measure dietary ALA. We attempted to address potential misclassification of diet by cumulatively updating dietary ALA intake for participants who had 2 measures of dietary ALA and did not develop CHD during follow‐up. Moreover, because our findings indicate that neither diet nor plasma phospholipid ALA is associated with incident AF, it is unlikely that the null findings are a result of error in dietary measurement alone. In total, 24% of the study population developed AF during follow‐up. On the basis of these numbers of events, we had 80% power to detect hazard ratios of 0.73 and 0.78 per standard deviation difference in plasma phospholipid and dietary ALA, respectively. Although we used both ECGs and hospital‐discharge diagnoses to identify incident AF, newly recognized paroxysmal AF that was managed in the outpatient setting would be missed. We adjusted for several factors known to be associated with ALA and AF, but it is possible that there may be residual confounding because of other unmeasured or poorly measured factors associated with ALA and AF. In the CHS, plasma phospholipid ALA levels were low and very similar to levels reported in several other cohorts, including ARIC, CARDIA, MESA, and the Physicians Health Study.^33–35^ Finally, our cohort comprised participants aged 65 years or older, and results may not be generalizable to younger populations.

## Conclusions

Among individuals consuming a typical American diet, neither ALA consumption (as assessed by FFQ) nor plasma ALA level was associated with AF. Additional studies are needed to investigate the association of ALA on AF in populations with higher ALA intake and different background diets, including diets low in LA, diets with no fish intake, and vegetarian diets.
